# Association of a novel meibomian gland dysfunction composite score with corneal nerve parameters and ocular surface symptoms in dry eye disease

**DOI:** 10.3389/fmed.2026.1877511

**Published:** 2026-07-10

**Authors:** Yao Yao, Zhaoxiang Xu, Yanhao Ma, Lijin Cui, Xiaotong Ren, Jian Guo

**Affiliations:** 1 Department of Ophthalmology, The First Affiliated Hospital of Fujian Medical University, Fuzhou, Fujian, China; 2 Department of Ophthalmology, National Regional Medical Center, Binhai Campus of the First Affiliated Hospital, Fujian Medical University, Fuzhou, Fujian, China; 3 Fujian Institute of Ophthalmology, The First Affiliated Hospital of Fujian Medical University, Fuzhou, Fujian, China; 4 Nanping Wuyi Housheng Eye Hospital, Nanping, China

**Keywords:** corneal nerves, dry eye disease, *in vivo* confocal microscopy, meibomian gland dysfunction, MGD composite score, symptom-sign discordance

## Abstract

**Purpose:**

To construct a composite meibomian gland dysfunction (MGD) score integrating gland dropout, expressibility, and meibum quality, and to investigate its associations with corneal nerve parameters, subjective symptoms, and ocular surface signs in patients with dry eye disease (DED).

**Methods:**

This cross-sectional study included 137 DED patients (274 eyes). MGD composite score (0–9) was calculated as the sum of averaged upper/lower eyelid scores for dropout (0–3), expressibility (0–3), and meibum quality (0–3). Corneal nerve parameters (length, width, reflectivity, microneuroma count) were assessed by *in vivo* confocal microscopy. Symptoms were evaluated using OSDI and a 10-item visual analogue scale. Tear break-up time (TBUT), corneal fluorescein staining (CFS), and tear meniscus height (TMH) were recorded. Correlation, partial correlation (to explore the associational pathway), and subgroup analyses were performed.

**Results:**

MGD composite score positively correlated with OSDI (*r* = 0.769, *p* < 0.01) and negatively with corneal nerve fiber length (CNFL) (*r* = −0.709, *p* < 0.01). OSDI correlated strongly with CNFL (*r* = −0.868, *p* < 0.01). After controlling for nerve length, age, sex, and CFS, the partial correlation between MGD composite score and OSDI dropped from 0.796 to 0.390. Patients with higher MGD grades exhibited significantly shorter nerve length, more microneuromas, worse TBUT, higher CFS, and greater symptom scores (all *p* < 0.05). Microneuroma count correlated positively with pain, asthenopia, blurred vision, and photophobia.

**Conclusion:**

The MGD composite score effectively reflects disease severity. Corneal nerve length is strongly associated with the relationship between MGD signs and subjective symptoms, providing a structural basis for understanding symptom-sign discordance in DED.

## 1 Introduction

Dry eye disease (DED) is a multifactorial disorder of the ocular surface characterized by loss of tear film homeostasis, accompanied by a spectrum of symptoms including dryness, pain, photophobia, and visual disturbances (1). The latest Tear Film and Ocular Surface Society (TFOS) Dry Eye Workshop (DEWS) III has refined the definition and classification of DED, emphasizing the central roles of inflammation, neurosensory abnormalities, and meibomian gland dysfunction (MGD) (2, 3). With a global prevalence of 5–50% and over 20% in China, DED imposes a substantial burden on patients’ quality of life and healthcare systems (4, 5).

MGD is the leading cause of evaporative dry eye, affecting approximately 70% of DED patients (6). It involves three core abnormalities: morphological (gland dropout), functional (reduced expressibility), and qualitative (altered meibum consistency) (7, 8). Recent advances in deep learning have enabled automated segmentation and quantitative analysis of meibomian gland morphology (9). While individual grading scales exist for each dimension (10, 11), a simple composite score that integrates all three aspects has been advocated but not systematically validated against objective measures of corneal nerve microstructure. Such a score could provide a more comprehensive assessment of MGD severity in both clinical practice and research.

Corneal nerves are essential for maintaining ocular surface integrity, regulating tear secretion, and sensing environmental stimuli. *In vivo* confocal microscopy (IVCM) enables non-invasive quantification of subbasal nerve parameters, including nerve fiber length, width, reflectivity, and the presence of microneuroma- also called corneal neuromas, bead-like terminal enlargements thought to represent aberrant nerve regeneration (12, 13). Previous studies have reported reduced corneal nerve density and increased microneuroma formation in DED patients, and these alterations correlate with symptom severity, particularly pain and photophobia (14, 15). However, whether a composite MGD score correlates with these nerve parameters in a dose-dependent manner has not been established.

A well-recognized clinical challenge in DED is the frequent discordance between objective signs and patient-reported symptoms (16, 17). Corneal nerve abnormalities are hypothesized to underlie this phenomenon (18, 19), but direct evidence linking MGD severity, nerve morphology, and symptom burden is lacking. Specifically, it remains unclear whether corneal nerve parameters mediate the relationship between MGD signs and subjective complaints.

Therefore, the present study aimed to: (1) construct a composite MGD score based on gland dropout, expressibility, and meibum quality; (2) investigate its associations with corneal nerve parameters, ocular surface symptoms and signs; (3) explore whether corneal nerve parameters are associated with the relationship between MGD severity and subjective symptoms using partial correlation analysis.

## 2 Methods

### 2.1 Study design and participants

This cross-sectional study was conducted using baseline data from a prospective randomized controlled trial. The study adhered to the Declaration of Helsinki and was approved by the Ethics Committee of the First Affiliated Hospital of Fujian Medical University (approval No. MTCA, ECFAH of FMU [2015]084–2). All participants provided written informed consent.

A total of 137 patients (274 eyes) diagnosed with DED (1) at The First Affiliated Hospital of Fujian Medical University (January–April 2025) were enrolled. Exclusion criteria: age>18 or <80 years; other ocular diseases (e.g., glaucoma, uveitis, keratitis), ocular surgery within 3 months, contact lens wear, systemic diseases affecting corneal nerves (e.g., diabetes mellitus, Parkinson’s disease, vitamin A deficiency), pregnancy or lactation.

### 2.2 MGD composite score

All eyelid assessments were performed by a single experienced ophthalmologist under a slit lamp.

Expressibility (obstruction) was assessed by applying digital pressure over the central five glands of each eyelid (7). Scores: 0, all glands expressible; 1, 3–4 glands expressible; 2, 1–2 glands expressible; 3, no gland expressible. Average of upper and lower eyelids was used.

Meibum quality was scored 0–3 for eight glands in the central one-third of each eyelid (7): 0, clear liquid; 1, cloudy liquid; 2, cloudy with granules; 3, toothpaste-like solid. Average of upper and lower eyelids was used.

Meibomian gland dropout was evaluated using infrared meibography (Oculus Keratograph 5 M, Germany). Each eyelid was scored 0–3: 0, no dropout; 1, dropout <1/3 of total gland area; 2, dropout 1/3–2/3; 3, dropout >2/3 (10). The final score was the average of upper and lower eyelids.

The MGD composite score was calculated as the sum of the three averaged scores (range 0–9, non-integer possible). Patients were classified into three severity grades: mild (0 < score ≤3), moderate (3 < score ≤6), and severe (6 < score ≤9).

### 2.3 Corneal nerve parameters by IVCM

IVCM was performed using a Heidelberg Retina Tomograph III with Rostock Cornea Module (Heidelberg Engineering, Germany). After topical anesthesia with 0.4% oxybuprocaine, the central cornea was scanned at the subbasal nerve plexus level (40–60 μm depth). Three high-quality, non-overlapping images per eye were selected for analysis by an examiner (RXT) masked to clinical data. “High-quality” was defined as clear focus, no motion artifact, good contrast, and <10% overlapping vessels. To assess interobserver repeatability, 20 randomly selected images were independently analyzed by two masked examiners, yielding an intraclass correlation coefficient (ICC) of 0.91 for CNFL. For image analysis, a semi-automated nerve tracing plugin in ImageJ software (National Institutes of Health, USA) was used, followed by manual correction by a single masked examiner (XZX) to ensure accuracy. Using this approach, we measured: corneal nerve fiber length (CNFL, μm per 400 × 400 μm field); corneal nerve fiber width (CNFW, μm); corneal nerve fiber reflectivity (CNFR, grey values, 0–255); and Microneuroma count —defined as bead-like terminal enlargements ≥5 μm in diameter, consistent with previously published criteria (20, 21) —per field. The average of three images was used for statistical analysis.

### 2.4 Subjective symptom assessment

OSDI was administered to assess overall dry eye-related quality of life (22). Scores range 0–100, with higher scores indicating worse symptoms.

A 10-item visual analogue scale (VAS, 0–10) was used to score individual symptoms (23, 24): dryness, foreign body sensation, pain, burning, watering, asthenopia, blurred vision, itching, increased secretions, and photophobia. A total symptom score (range 0–100) was calculated as the sum of the 10 items.

### 2.5 Ocular surface signs

Tear break-up time (TBUT): measured after instillation of 1 μL fluorescein sodium; the average of three measurements was recorded.

Corneal fluorescein staining (CFS): scored using the van Bijsterveld system (0–12) (25), dividing the cornea into four quadrants. CFS was graded by a single experienced observer (RXT) to ensure consistency.

Tear meniscus height (TMH): measured by the Oculus Keratograph in the central lower lid margin. Recent multicenter studies have validated the use of artificial intelligence-assisted techniques for objective tear meniscus height measurement, demonstrating high reproducibility and consistency across different clinical settings (26).

### 2.6 Statistical analysis

Analyses were performed using SPSS 26.0 (IBM Corp., Armonk, NY, USA). For each patient, values from both eyes were averaged to obtain a single patient-level measurement for all eye-specific parameters (e.g., MGD composite score, corneal nerve parameters, CFS). Normality was tested by Kolmogorov–Smirnov test. Data are presented as mean ± SD. For bivariate correlations, Pearson or Spearman coefficients were used as appropriate. Pearson or Spearman correlation analyses were then performed on these patient-level averaged data. Partial correlation analysis was performed to examine the association between MGD composite score and OSDI after sequentially adjusting for corneal nerve length, age, sex, and CFS. Patients were divided into three MGD severity groups (mild, moderate, severe); comparisons were made using ANOVA or Kruskal-Wallis with Bonferroni correction for post-hoc pairwise comparisons. All tests were two-sided, and *p* < 0.05 was considered statistically significant.

## 3 Results

### 3.1 General characteristics

As shown in Table 1, a total of 137 patients (274 eyes) were included, with a mean age of 53.08 ± 16.15 years (range 21–80). The cohort comprised 48 males and 89 females. For all subsequent correlation and comparative analyses, patient-level averaged values (derived from both eyes) were used (*n* = 137). The mean OSDI score was 43.49 ± 18.65.

**Table 1:** General information of the patients.

General information	Results
Number (people)	137
Number (eyes)	274
Sex (Male/female)	48/89
Age (Years, Mean ± SD, range)	53.08 ± 16.15(21–80)
OSDI (Scores, Mean ± SD, range)	43.49 ± 18.65(13–81)

All subsequent correlation and comparative analyses were performed on patient-level averaged data (*n* = 137).

### 3.2 Correlations between MGD composite score and other variables

#### 3.2.1 Bivariate correlation matrix

In Table 2, the MGD composite score was significantly positively correlated with OSDI (*r* = 0.769, 95% CI: 0.707 to 0.820, *p* < 0.01), CFS (*r* = 0.455, *p* < 0.01), microneuromas count (*r* = 0.536, *p* < 0.01), and negatively correlated with corneal nerve fiber length (*r* = −0.709, *p* < 0.01) and width (*r* = −0.299, *p* < 0.01). OSDI showed a strong negative correlation with nerve length (*r* = −0.868, 95% CI: −0.898 to −0.830, *p* < 0.01) and a positive correlation with microneuromas count (*r* = 0.664, *p* < 0.01). Nerve reflectivity was not significantly correlated with any of the other variables (all *p* > 0.05).

**Table 2:** Simple correlation coefficient matrix of MGD composite score and other variables.

	MGD Composite Score	OSDI	CFS	Microneuromas (N)	CNFL (μm)	CNFR (grey value)	CNFW (μm)
MGD composite score	1						
OSDI	0.769^**^	1					
CFS	0.455^**^	0.512^**^	1				
Microneuroma count	0.536^**^	0.664^**^	0.235^**^	1			
CNFL (μm)	−0.709^**^	−0.868^**^	−0.406^**^	−0.657^**^	1		
CNFR (grey values)	0.023	0.048	−0.011	0.001	−0.102	1	
CNFW (μm)	−0.299^**^	−0.240^**^	−0.126	−0.037	0.251^**^	−0.088	1

CFS, corneal fluorescein staining; CNFL, Corneal nerve fiber length; CNFW, Corneal nerve fiber width; CNFR, Corneal nerve fiber reflectivity. *p < 0.05; **p < 0.01.

#### 3.2.2 Partial correlation analysis

As shown in Table 3, after controlling for corneal nerve length, the partial correlation between MGD score and OSDI dropped from 0.796 to 0.439. Further adjustment for age and sex slightly reduced it to 0.415, and additional adjustment for CFS gave a partial correlation of 0.390 (all *p* < 0.001). This progressive decline suggests that corneal nerve length is a major mediator of the relationship between MGD severity and subjective symptoms.

**Table 3:** Correlation and partial correlation analysis of MGD composite score and OSDI.

Models	Control variable	Correlation coefficient	*p*
Model 1	None	*r* = 0.796	<0.001
Model 2	CNFL	*r* = 0.439	<0.001
Model 3	CNFL + age + sex	*r* = 0.415	<0.001
Model 4	CNFL + age + sex+ CFS	*r* = 0.390	<0.001

CFS, corneal fluorescein staining; CNFL, Corneal nerve fiber length.

### 3.3 Associations between corneal nerve parameters and symptoms

The correlations between corneal nerve parameters and ocular symptoms are summarized in Table 4. Microneuromas count was positively correlated with pain (*r* = 0.368, *p* = 0.016), asthenopia (*r* = 0.200, *p* = 0.004), blurred vision (*r* = 0.166, *p* = 0.018), and photophobia (*r* = 0.232, *p* = 0.001). Corneal nerve fiber length was negatively correlated with pain (*r* = −0.305, *p* = 0.043) and photophobia (*r* = −0.170, *p* = 0.017). Nerve fiber reflectivity was positively correlated only with foreign body sensation (*r* = 0.149, *p* = 0.037). No significant correlations were found for dryness, burning, watering, itching, or increased secretions.

**Table 4:** Correlation between corneal nerve parameters and ocular symptoms.

Symptom	Microneuroma count	CNFL	CNFR	CNFW
Dryness				
*r*	−0.115	−0.046	0.015	0.060
*p*	0.102	0.526	0.829	0.461
Foreign body sensation				
*r*	−0.025	−0.039	0.149*	0.038
*p*	0.727	0.586	0.037	0.636
Pain				
*r*	0.368*	−0.305*	−0.032	0.066
*p*	0.016	0.043	0.659	0.413
Burning				
*r*	−0.014	0.030	0.027	−0.086
*p*	0.846	0.676	0.708	0.286
Watering				
*r*	−0.047	−0.078	0.021	−0.110
*p*	0.503	0.276	0.771	0.175
Asthenopia				
*r*	0.200**	0.017	0.025	−0.044
*p*	0.004	0.813	0.722	0.588
Blurred vision				
*r*	0.166*	−0.032	0.001	−0.125
*p*	0.018	0.656	0.993	0.122
Itching				
*r*	0.016	−0.059	0.130	−0.118
*p*	0.817	0.411	0.069	0.144
Increased secretions				
*r*	0.126	−0.031	0.058	−0.091
*p*	0.072	0.664	0.415	0.263
Photophobia				
*r*	0.232**	−0.170*	0.087	−0.118
*p*	0.001	0.017	0.222	0.144
Total score				
*r*	−0.119	−0.073	0.086	−0.104
*p*	0.089	0.310	0.227	0.199

CNFL, Corneal nerve fiber length; CNFW, Corneal nerve fiber width; CNFR, Corneal nerve fiber reflectivity. *p < 0.05; **p < 0.01. The bolded indicate statistical significance.

### 3.4 Associations between corneal nerve parameters and signs

The correlations between corneal subbasal nerve parameters and ocular signs are presented in Table 5. Microneuroma count showed modest positive correlations with meibum quality scores of both upper (*r* = 0.140, *p* = 0.045) and lower (*r* = 0.151, *p* = 0.031) eyelids. Corner nerve fiber length also correlated positively with meibum quality (upper: *r* = 0.165, *p* = 0.021; lower: *r* = 0.147, *p* = 0.039). Corneal nerve fiber reflectivity was weakly positively correlated with TMH (*r* = 0.164, *p* = 0.022), but not with TBUT or MG obstruction scores (all *p* > 0.05). Nerve width was not significantly associated with any sign. Of note, the weak positive correlations between CNFL and meibum quality (*r* ≈ 0.15, *p* < 0.05) appear counterintuitive. Given the restricted range of meibum quality scores and the negligible explained variance (<2%), these findings are unlikely to be clinically meaningful and may represent statistical artifact.

**Table 5:** Correlation between corneal nerve parameters and ocular signs.

Signs	Microneuroma count	CNFL	CNFW	CNFR
TMH (mm)				
*r*	−0.007	0.027	0.011	0.164*
*p*	0.921	0.705	0.896	0.022
TBUT(s)				
*r*	0.026	−0.054	−0.095	−0.045
*p*	0.714	0.452	0.238	0.529
Expressibility (upper, 0–9)				
*r*	0.116	−0.004	0.105	0.007
*p*	0.097	0.957	0.194	0.924
Expressibility (lower, 0–9)				
*r*	0.107	0.023	0.104	0.013
*p*	0.129	0.750	0.196	0.856
Secretion quality(upper, 0–3)				
*r*	0.140*	0.165*	0.103	0.035
*p*	0.045	0.021	0.203	0.624
Secretion quality(lower, 0–3)				
*r*	0.151*	0.147*	0.105	0.025
*p*	0.031	0.039	0.195	0.729

CNFL, Corneal nerve fiber length; CNFW, Corneal nerve fiber width; CNFR, Corneal nerve fiber reflectivity; TMH, tear meniscus height; TBUT, tear film break-up time; CFS, corneal fluorescein staining. *p < 0.05. The weak positive correlations between CNFL and meibum quality (*r* ≈ 0.15) are not clinically meaningful and may reflect statistical artifact due to restricted score range. The bolded indicate statistical significance.

### 3.5 Comparisons across MGD severity groups

Patients were categorized into mild (*n* = 23, score ≤3), moderate (*n* = 42, 3 < score≤6), and severe (*n* = 72, score>6) MGD groups. Age and sex did not differ among groups (*p* > 0.05). Table 6 presents the symptoms, signs and corneal nerve parameters of different MGD grades.

**Table 6:** Symptoms, signs, and corneal nerve parameters of different MGD grades.

		Mild group	Moderate group	Severe group	P1	P2	P3
General information	Number (People)	23	42	72			
	Sex (M/F)	8/15	15/27	25/47			
	Age (Years)	51.65 ± 11.86	53.40 ± 17.69	55.94 ± 15.47	0.239	0.107	0.445
Symptoms (scores)	Dryness	4.39 ± 2.92	5.70 ± 2.77	5.82 ± 3.08	0.261	0.125	0.753
	Foreign body sensation	2.57 ± 1.44	4.80 ± 3.47	4.63 ± 3.19	**0.018**	**0.017**	0.522
	Pain	1.39 ± 2.89	3.35 ± 3.01	5.49 ± 3.23	0.053	**<0.001**	**0.002**
	Burning	0.91 ± 1.44	1.43 ± 1.97	2.07 ± 3.12	0.358	0.195	0.630
	Watering	1.65 ± 2.12	2.53 ± 2.90	4.01 ± 3.36	0.827	**0.005**	**0.044**
	Asthenopia	4.43 ± 2.27	5.13 ± 2.93	6.15 ± 3.54	0.201	0.078	0.313
	Blurred vision	2.00 ± 2.11	3.00 ± 2.60	4.22 ± 3.63	0.676	**0.011**	0.151
	Itching	1.09 ± 1.70	2.78 ± 2.17	2.93 ± 2.74	**0.027**	**0.006**	0.654
	Increased secretions	2.26 ± 2.32	3.20 ± 3.15	2.75 ± 2.90	0.648	0.712	0.251
	Photophobia	0.01 ± 0.01	3.65 ± 3.14	7.28 ± 2.38	**<0.001**	**<0.001**	**<0.001**
	Total	24.96 ± 7.54	38.28 ± 13.35	45.14 ± 14.85	**<0.001**	**<0.001**	**<0.001**
	OSDI	20.60 ± 5.13	35.36 ± 9.95	55.32 ± 15.64	**<0.001**	**<0.001**	**<0.001**
Signs	TMH(mm)	0.19 ± 0.05	0.16 ± 0.05	0.14 ± 0.05	**0.034**	**<0.001**	0.537
	TBUT(s)	8.17 ± 1.97	4.47 ± 1.58	3.86 ± 1.14	**<0.001**	**<0.001**	0.100
	CFS	0.04 ± 0.20	1.70 ± 2.55	3.71 ± 3.45	0.231	**<0.001**	**0.014**
Corneal nerve parameters	Microneuroma count	0.26 ± 0.45	0.95 ± 1.30	2.10 ± 0.51	0.148	**<0.001**	**<0.001**
	CNFL (μm)	2648.48 ± 368.69	1867.74 ± 672.47	1151.96 ± 554.32	**<0.001**	**<0.001**	**<0.001**
	CNFR (grey values)	129.59 ± 16.23	141.25 ± 24.76	138.68 ± 19.07	0.095	0.200	0.476
	CNFW(μm)	8.79 ± 4.79	5.85 ± 1.42	5.51 ± 1.22	0.087	0.076	0.643

TMH, tear meniscus height; TBUT, tear film break-up time; CFS, corneal fluorescein staining; CNFL, Corneal nerve fiber length; CNFW, Corneal nerve fiber width; CNFR, Corneal nerve fiber reflectivity. The bolded indicate statistical significance. The MGD composite score was calculated as the sum of the three averaged scores (range 0–9, non-integer possible). Patients were classified into three severity grades: mild (0 < score ≤3), moderate (3 < score ≤6), and severe (6 < score ≤9). P1: Mild group vs. Moderate group; P2: Mild group vs. Severe group; P3: Moderate group vs. Severe group.

Symptoms: OSDI progressively increased across groups (20.60 ± 5.13 vs. 35.36 ± 9.95 vs. 55.32 ± 15.64, all pairwise *p* < 0.001). Total symptom score, photophobia, pain, and watering were significantly higher in the severe group compared to mild and moderate groups (all *p* < 0.05). Blurred vision and itching showed significantly higher in the severe group compared to moderate groups (all *p* < 0.05). Dryness, burning, asthenopia, and increased secretions did not differ significantly across groups.

Signs: TMH and TBUT in severe showed significantly decreased compared to mild and moderate (all *p* < 0.05), but there were no significant difference between moderate and severe groups (all *p* > 0.05). CFS increased (0.04 ± 0.20 vs. 1.70 ± 2.55 vs. 3.71 ± 3.45, *p* < 0.001 for mild vs. severe, *p* = 0.014 for moderate vs. severe).

**Figure 1:**
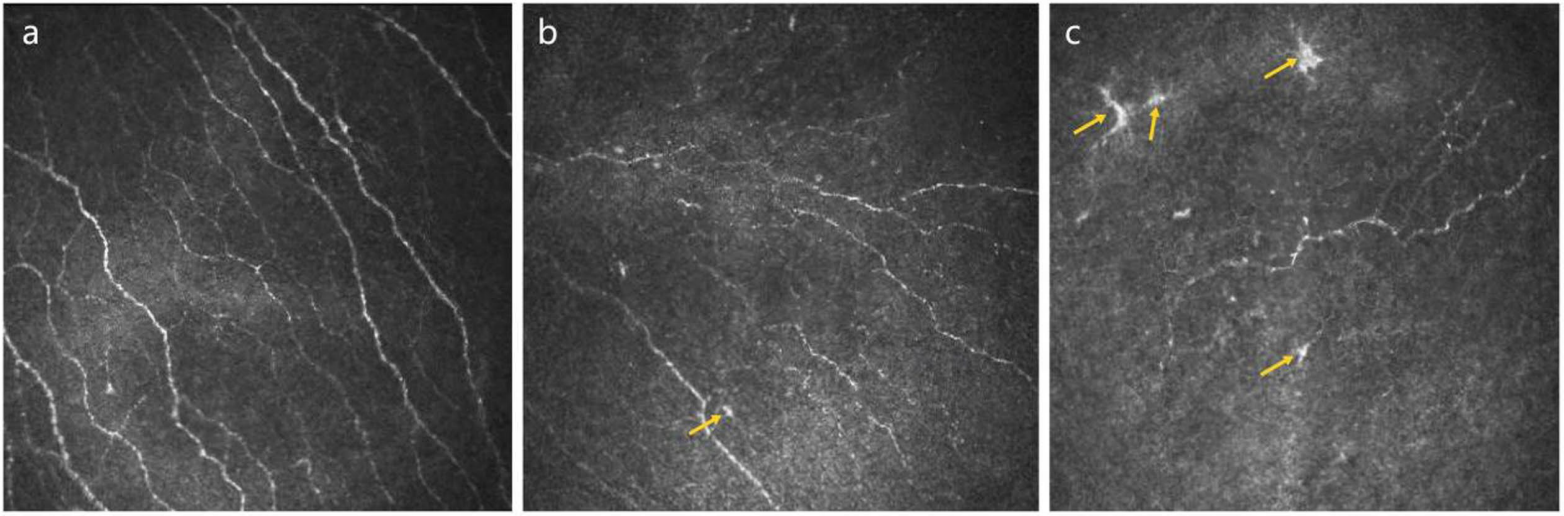
Representative in vivo confocal microscopy (IVCM) images of the subbasal corneal nerve plexus across MGD severity grades. **(a)** Mild MGD (composite score ≤3): dense corneal nerve fiber network with few microneuromas. **(b)** Moderate MGD (3 < score ≤6): reduced nerve fiber density with occasional bead-like microneuromas (arrows). **(c)** Severe MGD (score >6): markedly reduced nerve fiber length with multiple microneuromas (arrows).

Corneal nerve parameters: Nerve firber length shortened dramatically from mild to severe (2648.48 ± 368.69 μm vs. 1867.74 ± 672.47 μm vs. 1151.96 ± 554.32 μm, all pairwise *p* < 0.001). Microneuroma number increased (0.26 ± 0.45 vs. 0.95 ± 1.30 vs. 2.10 ± 0.51, *p* < 0.001 for mild vs. severe and moderate vs. severe; mild vs. moderate *p* = 0.148). Nerve fiber width and reflectivity showed no significant group differences. Representative IVCM images from patients with mild, moderate, and severe MGD are shown in Figure 1, illustrating the progressive reduction in corneal nerve fiber length and the corresponding increase in microneuroma count across severity grades.

The MGD composite score was calculated as the sum of the three averaged scores (range 0–9, non-integer possible). Patients were classified into three severity grades: mild (0 < score ≤3), moderate (3 < score ≤6), and severe (6 < score ≤9). P1: Mild group vs. Moderate group; P2: Mild group vs. Severe group; P3: Moderate group vs. Severe group.

## 4 Discussion

### 4.1 Principal findings

This study developed a novel composite MGD score that integrates gland dropout, expressibility, and meibum quality, and systematically examined its associations with corneal nerve parameters, symptoms, and signs in a large DED cohort. Three principal findings emerged. First, the MGD composite score showed strong correlations with established clinical tests (TBUT, CFS) and patient-complained symptoms, supporting its validity as a comprehensive severity measure. Second, corneal nerve length demonstrated a dose-dependent negative relationship with MGD severity, with severe MGD patients having nerve lengths less than half of those with mild MGD. Third, and most importantly, partial correlation analysis revealed that controlling for corneal nerve length reduced the MGD-OSDI correlation from 0.796 to 0.439, indicating that nerve length mediates a substantial portion of the relationship between MGD signs and subjective symptoms. These findings provide a structural basis for understanding the often-observed discordance between ocular surface signs and patient symptoms in DED (16, 17).

### 4.2 Validity and clinical utility of the MGD composite score

Although individual MGD grading systems have been well established for over a decade (7, 10), a composite score that captures both structural and functional abnormalities has been recommended but never systematically validated against corneal nerve parameters. Our MGD composite score (0–9) is simple to calculate in routine practice, requiring only slit-lamp examination and meibography. Its strong correlations with TBUT (inversely), CFS (positively), and OSDI (positively) confirm that it reflects clinically meaningful disease burden. Notably, the score discriminated well among the three severity groups, with significant stepwise differences in TBUT, CFS, and OSDI. This is consistent with the TFOS DEWS III recommendation that MGD assessment should integrate multiple dimensions (3, 8). Compared to using only meiboscore or expressibility alone, the composite score likely provides a more holistic picture of MGD and may be more sensitive to treatment-induced changes in longitudinal studies. We acknowledge that the equal weighting of the three MGD components was an arbitrary choice made for clinical simplicity and consistency with prior TFOS frameworks. To explore whether individual components differ in their associations with clinical outcomes, we performed an exploratory analysis comparing the composite score with each component separately. The composite score showed higher correlations with OSDI (*r* = 0.769) than meiboscore alone (*r* = 0.612), expressibility alone (*r* = 0.541), or meibum quality alone (*r* = 0.498), and similarly stronger correlations with CNFL (−0.709 vs. −0.548, −0.503, and −0.461, respectively). These findings suggest that the composite score provides additional explanatory power beyond any single component, though we acknowledge that differential weighting might further optimize its performance. Future research could explore whether weighting the three components differently improves its predictive value for specific symptoms (e.g., pain, dryness). The relatively strong correlation between MGD composite score and OSDI in our cohort (*r* = 0.769) warrants explanation. Several factors may contribute: our composite score integrates three distinct dimensions of MGD, capturing a broader spectrum of glandular pathology than any single parameter; the high proportion of moderate-to-severe MGD (72/137, 52.6%) widened the severity range; and the use of patient-level averaged data reduced measurement noise. These factors collectively may have enhanced the correlation relative to studies using single-parameter assessments.

### 4.3 Dose-dependent relationship between MGD severity and corneal nerve damage

Our subgroup analysis revealed a striking dose-dependent association: as MGD severity increased from mild to severe, corneal nerve length decreased from 2,648 μm to 1,152 μm — a reduction of nearly 57%. This is, to our knowledge, the first demonstration of such a clear gradient between a standardized MGD composite score and IVCM-derived nerve length. Previous studies have reported reduced corneal nerve density in DED compared to healthy controls (12, 13), but few have stratified by MGD severity within a DED population. Cox et al. (27) compared aqueous deficient and evaporative dry eye subtypes and found more severe corneal nerve loss in the former, but they did not use a composite MGD score or examine dose-dependency. Our data suggest that the cumulative inflammatory and mechanical stress from chronically obstructed and abnormal meibum is associated with progressively damage corneal nerve fibers. The parallel increase in microneuroma count (0.26 to 0.95 to 2.10) indicates that nerve degeneration is accompanied by abnormal regenerative attempts, which may be associated with neuropathic symptoms (28–30). Interestingly, nerve width and reflectivity did not differ significantly across groups. While some studies have reported increased nerve reflectivity in DED as a sign of axonal degeneration (31), our results suggest that width and reflectivity might be more variable or influenced by imaging factors, and length may be a more robust metric for MGD-related nerve damage.

Whether corneal nerve shortening in MGD is reversible remains incompletely understood. However, emerging evidence suggests that effective MGD treatment may promote corneal nerve regeneration. In a prospective interventional study from our group, intense pulsed light (IPL) combined with meibomian gland expression significantly increased corneal nerve fiber length (CNFL) from 1.82 ± 0.68 mm to 2.45 ± 0.65 mm after three treatment sessions (*p* < 0.001) (32). This finding indicates that at least a component of MGD-associated nerve damage may be reversible with appropriate therapy. Nevertheless, the extent of reversibility likely depends on the duration and severity of MGD; chronic, longstanding glandular obstruction may lead to more permanent structural alterations. Longitudinal studies tracking nerve changes after treatment are needed to determine the time course and predictors of nerve recovery.

### 4.4 Corneal nerve fiber length as a mediator of the MGD-symptom relationship: implications for symptom-sign discordance

A central finding of this study is the strong negative correlation between OSDI and corneal nerve length (*r* = −0.868), which was even stronger than the correlation between MGD score and OSDI (*r* = 0.769). However, the cross-sectional design of this study precludes definitive conclusions about the direction of causality. While severe MGD may lead to corneal nerve damage through chronic inflammation, mechanical stress, and abnormal meibum toxicity, it is equally plausible that pre-existing variations in corneal nerve density or function influence symptom perception and disease progression. This bidirectional relationship—whereby MGD and nerve pathology may reinforce each other—is consistent with the vicious cycle model of DED pathophysiology (8, 18). The partial correlation analysis further demonstrated that after controlling for nerve length, the direct association between MGD score and OSDI dropped from 0.796 to 0.439 — a 45% reduction. When age, sex, and CFS were additionally controlled, the correlation fell further to 0.390. This pattern indicates that a substantial portion of the impact of MGD on subjective symptoms is mediated through corneal nerve damage, rather than being a direct effect of the glandular abnormality itself.

This pattern suggests that a substantial portion of the association between MGD and subjective symptoms is related to corneal nerve status, providing a structural explanation for the well-known phenomenon of symptom-sign discordance in DED (16, 17). Even though our study population showed an overall high correlation between MGD signs and symptoms (*r* = 0.769), meaning that most patients with severe MGD also reported severe symptoms, the mediation analysis reveals that two patients with identical MGD scores could experience vastly different symptom levels depending on the integrity of their corneal nerves. In other words, the corneal nerve status is a critical “filter” that determines how much of the peripheral glandular pathology translates into conscious discomfort. This is consistent with the TFOS DEWS III pain and sensation report, which emphasizes that neurosensory abnormalities can amplify or dampen symptom perception independently of local tissue damage (18). For clinicians, this implies that when a patient’s symptoms are disproportionately severe relative to MGD signs, IVCM assessment of corneal nerve fiber length and microneuromas may help identify a neuropathic component that requires different management (e.g., nerve- regenerative therapies rather than solely gland-directed treatments) (33).

Nevertheless, a residual partial correlation of 0.390 remained after full adjustment, indicating that factors other than corneal nerve length also contribute to symptom generation. These may include tear inflammatory cytokines (e.g., IL-6, MMP-9) (34, 35), psychological distress, central sensitization, or other aspects of corneal nerve function not captured by morphological parameters (36).

### 4.5 Microneuromas as potential biomarkers of neuropathic symptoms

Microneuroma, also called corneal neuromas, are bead- like terminal enlargements of corneal nerve fibers that have been proposed as *in vivo* biomarkers of neuropathic corneal pain (20, 21). From a clinical perspective, IVCM-based microneuroma assessment may be particularly useful in patients whose symptoms appear disproportionate to ocular surface signs. In such cases, the presence of numerous microneuromas—especially when accompanied by reduced CNFL—could support the diagnosis of a neuropathic component and guide clinicians toward nerve-targeted therapies (e.g., vitamin B12, autologous serum tears, or neuroprotective agents) (23) rather than solely gland-directed interventions. However, prospective studies are needed to validate microneuroma assessment as a clinical decision-making tool. In our study, microneuroma count correlated positively with pain (*r* = 0.368), asthenopia (*r* = 0.200), blurred vision (*r* = 0.166), and photophobia (*r* = 0.232). Moreover, microneuroma count increased stepwise with MGD severity: from 0.26 in mild MGD to 0.95 in moderate and 2.10 in severe MGD. These observations support the concept that chronic MGD-induced inflammation and mechanical stress are associated not only with nerve fiber loss (shortened length) but also with trigger aberrant regenerative sprouting that manifests as microneuromas. Such microneuromas are thought to generate spontaneous or evoked pain due to ectopic discharge (37, 38).

Our findings align with those of Moein et al. (28), who reported that microneuroma visualization by IVCM had high sensitivity and specificity for diagnosing neuropathic corneal pain. However, unlike Dermer et al. (39) who found no difference in microneuroma counts between DED and controls, we observed a clear gradient across MGD severity groups. The discrepancy may be due to differences in patient selection: our cohort had a high proportion of moderate-to-severe MGD (72/137 severe), whereas Dermer’s study included a mix of DED subtypes with possibly milder MGD.

The correlation between microneuromas and photophobia (*r* = 0.232) is also clinically relevant. Photophobia is a common and disabling symptom in DED and is thought to involve trigeminal nerve sensitization (40). Our data suggest that microneuroma formation may contribute to photophobia via aberrant activation of trigeminal pain pathways. Future studies using quantitative sensory testing could further clarify this relationship.

### 4.6 Clinical implications, limitations, and future directions

The MGD composite score provides a quick, standardized tool for routine clinical assessment. Patients with moderate-to-severe scores should receive aggressive gland-directed therapies such as intense pulsed light or thermal pulsation (24, 32). More importantly, when symptoms are disproportionate to MGD signs, IVCM assessment of corneal nerve length and microneuromas can help identify a neuropathic component, guiding the use of nerve-regenerative treatments such as oral vitamin B12, autologous serum tears, or cenegermin (23, 33, 41). To operationalize this approach in clinical practice, we propose the following algorithm for patients whose symptom burden (e.g., OSDI > 40) appears disproportionate to the degree of MGD signs: (1) perform IVCM to assess CNFL and microneuroma count; (2) if CNFL is markedly reduced (<1,500 μm per field) or microneuroma count is elevated (>2 per field), this suggests a neuropathic component that may require nerve-targeted interventions (e.g., oral vitamin B1/mecobalamin, autologous serum tears); (3) if corneal nerve parameters are relatively preserved, consider alternative contributors to symptom generation, such as tear hyperosmolarity, inflammation, or central sensitization. This structured evaluation moves beyond the simplistic ‘signs versus symptoms’ dichotomy and aligns with the TFOS DEWS II framework that emphasizes neurosensory abnormalities as a core mechanism of DED. The strong correlation between nerve length and OSDI also suggests that promoting corneal nerve regeneration could be a therapeutic consideration even in patients without obvious neuropathy.

There were some limitations in this study. First, the absence of a healthy control group limits our ability to determine whether the observed corneal nerve parameters in DED patients are abnormal relative to normative values. While previous studies have established that DED patients have reduced corneal nerve density and increased microneuroma counts compared with healthy controls, the specific gradient of nerve loss across MGD severity grades observed in our cohort cannot be benchmarked against normative data without a control group. Future studies should include age- and sex-matched healthy controls to establish reference values. Second, the cross- sectional design precludes causal inferences. While our mediation analysis using partial correlations suggests a mediating role of nerve length, longitudinal studies tracking changes after MGD treatment are needed to confirm causality. Third, the equal weighting of the three MGD components may not reflect their true pathophysiological contributions. Some patients may have predominant dropout but good secretion, while others have severe obstruction without dropout; these different phenotypes might have different corneal nerve impacts. Fourth, the sample size was imbalanced across severity groups, with the mild group (*n* = 23) being substantially smaller than the severe group (*n* = 72). This imbalance may have reduced statistical power for subgroup comparisons and could influence the robustness of pairwise comparisons, particularly those involving the mild group. Future studies with balanced recruitment across severity grades are warranted. In addtion, this was a single-center study, and generalizability to other populations (e.g., older adults, different ethnicities) requires validation. Fifth, we did not measure tear inflammatory cytokines, which would have strengthened the mechanistic link between MGD and nerve damage. Sixth, the partial correlation approach suggests an associational pathway but does not constitute formal mediation analysis; future studies using bootstrapping-based mediation models are needed to test causal mediation. Seventh, we did not collect comprehensive data on potential confounders including duration of DED, systemic medications (e.g., antihistamines, antidepressants, hormonal therapies), hormonal status (e.g., menopausal status), autoimmune disease screening (e.g., anti-SSA/SSB antibodies, rheumatoid factor), or prior dry eye treatments other than artificial tears. These factors may influence corneal nerve morphology and symptom perception. We have acknowledged this as an important limitation and recommend that future studies systematically capture these variables to enable adjusted analyses. Eighth, the partial correlation approach suggests an associational pathway but does not constitute formal mediation analysis; future studies using bootstrapping-based mediation models are needed to test causal mediation.

In the future, longitudinal studies tracking corneal nerve changes after effective MGD treatment (e.g., IPL, LipiFlow) are needed. Larger multicenter cohorts should validate the optimal weighting of the composite score. Incorporating tear biomarkers could clarify the inflammatory pathway from MGD to neuropathy. Additionally, future studies should systematically collect data on potential confounders—including DED duration, systemic medications, hormonal status, and autoimmune screening—to enable adjusted analyses and more precisely isolate the independent contribution of MGD to corneal nerve alterations. The rapid growth of AI applications in ophthalmic imaging highlights the expanding potential for integrating multimodal clinical data into individualized treatment frameworks (42). Finally, interventional trials comparing gland-directed versus nerve- directed therapies in MGD patients with prominent neuropathic symptoms are warranted.

## 5 Conclusion

The novel MGD composite score (0–9) integrating gland dropout, expressibility, and meibum quality effectively reflects disease severity in DED, as evidenced by strong correlations with TBUT, CFS, and OSDI. Corneal nerve fiber length demonstrates a dose- dependent reduction across MGD severity grades and is strongly associated with the relationship between MGD signs and subjective symptoms, providing a structural basis for understanding symptom-sign discordance. Microneuroma count increases with MGD severity and correlates with pain, asthenopia, and photophobia, supporting its role as a potential biomarker of neuropathic symptoms. IVCM- derived corneal nerve parameters offer valuable complementary information for individualized management of DED patients, especially those with disproportionate symptom burden.

## Data Availability

The raw data supporting the conclusions of this article will be made available by the authors, without undue reservation.
