# Supplementary material for: Association of a novel meibomian gland dysfunction composite score with corneal nerve parameters and ocular surface symptoms in dry eye disease
Source: Front Med (Lausanne). 2026 Jul 10;13:1877511. doi: 10.3389/fmed.2026.1877511 (PMC13395767; doi:10.3389/fmed.2026.1877511)
Supplement: Supplementary file 1 [file Table_1.DOCX]

**Supplementary Table S1. Comparison of correlation coefficients between MGD composite score, individual components, and clinical outcomes**

| MGD severity measure | Correlation with OSDI (r) | Correlation with CNFL (r) |
| --- | --- | --- |
| MGD composite score | 0.769 | −0.709 |
| Dropout alone | 0.612 | −0.548 |
| Expressibility alone | 0.541 | −0.503 |
| Meibum quality alone | 0.498 | −0.461 |

All correlations were statistically significant (P < 0.01). Data were derived from patient-level averaged measurements (n=137). For each individual component, the score represents the average of upper and lower eyelid assessments, averaged across both eyes, consistent with the methodology used for the composite score.
